# Characterisation and antimicrobial susceptibility pattern of non-tuberculous mycobacteria

**DOI:** 10.4102/sajid.v39i1.525

**Published:** 2024-01-05

**Authors:** Abraham J. le Roux, Anneke van der Spoel van Dijk, Motlatji R.B. Maloba

**Affiliations:** 1 Department of Medical Microbiology, Faculty of Health Sciences, University of the Free State, Bloemfontein, South Africa

**Keywords:** non-tuberculous mycobacteria, *Mycobacteria avium* complex, *Mycobacterium abscessus*, GenoType^®^ NTM-DR, genotypic antimicrobial susceptibility

## Abstract

**Background:**

Non-tuberculous mycobacteria (NTM) management comprises prolonged therapy that includes macrolides. Non-tuberculous mycobacteria can cause disease in patients with predisposing conditions such as HIV and structural lung disease. Local data on NTM disease and macrolide resistance are scarce, and routine antimicrobial susceptibility testing is currently not performed for NTM in South Africa.

**Objectives:**

This study aims to characterise NTM isolated at Tshepong National Health Laboratory Service (NHLS) according to species and antimicrobial susceptibility pattern.

**Method:**

A retrospective data analysis of NTM isolates from Tshepong NHLS was performed from January to June 2020. GenoType^®^ NTM-DR was performed on selected isolates where the assay can confirm the species and determine resistance to macrolides and aminoglycosides.

**Results:**

Of the 194 collected NTM isolates, 183 were included in the study. Patients’ ages ranged from 1 day to 81 years (median 36 years). The most common specimen was sputum (84.7%), followed by gastric aspirate (6.6%). The most common NTM isolated were *Mycobacterium (M.) intracellulare* (67.6%), *M. fortuitum* (12.6%), *M. species* (4.3%), *M. kansasii* (3.9%), and *M. scrofulaceum* (3.9%). Macrolide resistance occurred in 2.8% of tested isolates; no aminoglycoside resistance was detected. Although most isolates were from males (62.3%), resistance was observed only in females.

**Conclusion:**

*M. intracellulare* predominated, with only two *M. intracellulare* and two *M. abscessus* isolates showing macrolide resistance; aminoglycoside resistance was absent.

**Contribution:**

This study highlights the need for increased awareness of NTM, regular nationwide NTM surveillance, and monitoring of resistance trends to guide future patient management and ensure good treatment outcomes.

## Introduction

Non-tuberculous mycobacteria (NTM) comprise an estimated 200 species of mycobacteria, excluding the *Mycobacterium tuberculosis* (MTB) complex and *M. leprae* complex.^1,2^ Although widely present in the environment, they can cause disease in patients with risk factors such as structural lung disease, advanced HIV, or penetrating skin and soft tissue injuries.^2,3^ Worldwide, NTM-related diseases appear to be increasing.^4^ Potential factors contributing to the rise in NTM lung disease include improved clinical awareness, improved diagnostics, greater exposure to environmental NTM, and patient factors such as increased rates of bronchiectasis, chronic obstructive pulmonary disease, or the use of immunosuppressive agents such as corticosteroids.^5^ Within the South African context, prevalent risk factors for NTM disease include advanced HIV, prior tuberculosis (TB), silicosis, and prolonged underground mining exposure.^6,7^ However, determining incidence and prevalence is challenging because NTM disease is typically not a notifiable medical condition. Additionally, the isolation of NTM, particularly from non-sterile sites, often indicates contamination or colonisation rather than active disease.^4,8^

Non-tuberculous mycobacteria infection can manifest as pulmonary disease (NTM-PD), lymphatic disease, skin and soft tissue infection, and disseminated disease, of which NTM-PD is the most common.^3^ The diagnosis of NTM-PD requires clinical, microbiological, and radiological criteria to be met, as most isolates are not clinically significant.^2,9^ It can also mimic pulmonary tuberculosis (PTB).^4^ From 2007 to 2008 in Burkina Faso, 20% of patients with presumed chronic PTB had NTM-PD.^10^ In the context of sub-Saharan Africa, a meta-analysis focusing on NTM isolated from respiratory samples unveiled a substantial prevalence of pulmonary NTM colonisation, reaching up to 7.5%.^11^ Moreover, a notable 27.9% of participants in the study satisfied the criteria for NTM-PD.^11^

Laboratory diagnosis of NTM can be lengthy as it is culture-based.^12^ Culture is often performed using a continuously monitored broth culture instrument system, which reduces incubation time.^12^ Microscopy with acid-fast stains may be employed for mycobacteria detection; however, its sensitivity is suboptimal and depends on bacterial load and the proficiency of the microscopist.^13^ Furthermore, it lacks specificity as other bacteria can also stain acid-fast. Importantly, it cannot reliably distinguish between NTM and MTB, nor can it determine viability.^12,14^ Previously, NTM were identified using a series of biochemical reactions, growth rate, and pigmentation of colonies, but this was time-consuming and not consistent.^12^ Now, molecular tests such as the GenoType^®^ Mycobacterium CM (Hain Lifescience, Germany) are widely used to identify NTM.^12^ This test is a multiplex polymerase chain reaction (PCR) in a line probe assay format that can detect the most common NTM.^15^

The treatment of NTM disease can be challenging as it requires a prolonged course of macrolide and aminoglycoside-containing combination therapy, sometimes in conjunction with surgical resection.^2^ Even if treated successfully, many patients have relapses or re-infection, particularly in cases of macrolide resistance.^2,16^ Park et al. conducted a meta-analysis to assess the outcomes of patients with pulmonary disease caused by macrolide-resistant *M. avium* complex (MAC). They found a modest 21% sputum culture conversion rate over 1 year, with a 1-year all-cause mortality rate of 10%.^17^ The increased risk of treatment failure in cases of macrolide resistance underscores the importance of investigating novel treatment modalities.^17^

Currently, susceptibility testing of NTM isolates is not routinely conducted, and only a limited number of laboratories have the capacity for such testing.^12^ However, it is important in MAC, *M. abscessus*, and *M. kansasii* isolates, given the direct relation between susceptibility results and clinical response.^12^ In treatment-naïve patients, the susceptibility of MAC to macrolides and aminoglycosides is typically observed.^18^ However, MAC resistance can occur in patients previously exposed to these drugs because of mutations in the *rrl* and *rrs* genes.^16,18,19^ In contrast, most *M. abscessus* species have inducible macrolide resistance mediated by a functional *erm* (41) gene.^20^ The gold standard for antimicrobial susceptibility testing is broth microdilution.^12^ However, it has numerous challenges, such as long turnaround times, antibiotic degradation, and difficult interpretation because of trailing endpoints.^12,21^ For these reasons, multiplex PCR-based molecular assays provide an attractive alternative.^21,22^ GenoType^®^ NTM-DR (Hain Lifescience, Germany) is one such method that can identify MAC (including *M. intracellulare*), *M. abscessus* complex, and *M. chelonae* species, as well as the detection of macrolide and aminoglycoside resistance mediated by the *rrl, erm*, and *rrs* genes.^23^ According to numerous studies, GenoType^®^ NTM-DR showed excellent concordance of more than 90% when compared to both broth microdilution and sequencing.^21,24,25^ However, it has limitations; it can only detect resistance mediated by the specific *erm*(41), *rrl*, and *rrs* gene regions.^23^

Numerous published studies performed in South Africa have investigated NTM, but to the best of our knowledge, none of the published studies in South Africa have investigated NTM drug resistance.^26,27,28,29,30^

The study aimed to characterise previously stored NTM isolates at Tshepong National Health Laboratory Service (NHLS) and to determine their susceptibility to macrolides and aminoglycosides.

## Research methods and design

### Study design and setting

This quantitative, retrospective, descriptive study was conducted at NHLS Universitas Academic Hospital Laboratory. The study was performed on NTM isolates from Tshepong NHLS in Klerksdorp in the North West province. Tshepong NHLS is a referral laboratory that services clinics and hospitals in Dr Kenneth Kaunda District as well as the Dr Ruth Segomotso Mompati District, including Joe Morolong Hospital, Taung Hospital, Ganyessa Hospital, Chriatiana Hospital, Schweizer-Reneke Hospital, Tshepong Hospital, Potchefstroom Hospital, and Nic Bodenstein Hospital. These healthcare facilities predominantly serve the agricultural and mining industry community, including people living in informal settlements.^31,32^

### Study population and sampling strategy

**FIGURE 1:**
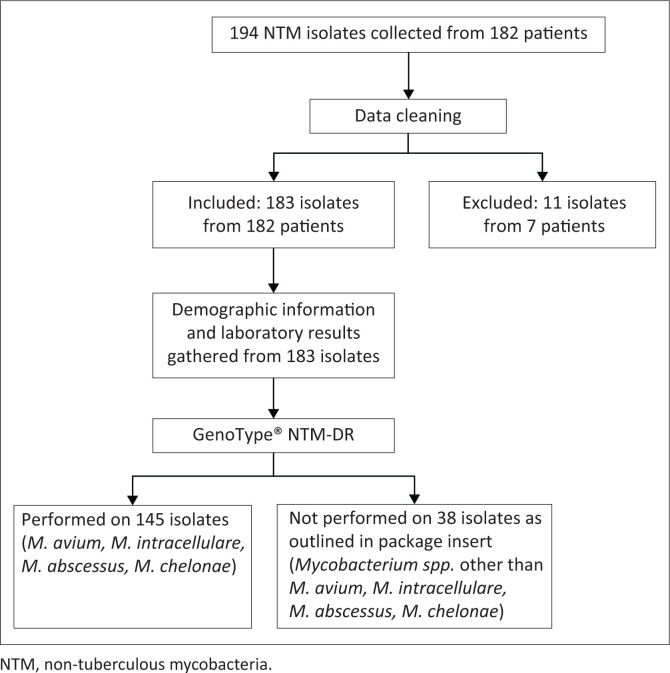
Flow diagram summarising non-tuberculous mycobacteria isolates characterised using GenoType CM, followed by speciation and resistance testing using GenoType NTM-DR for identified isolates within the *M. avium* complex, *M. abscessus* complex, and *M. chelonae.*

The convenience sampling method was used. Stored NTM isolated from specimens that were submitted for routine TB investigations (auramine staining and TB culture) from January 2020 to June 2020 were obtained from Tshepong NHLS. All specimens were subjected to auramine staining, followed by culture using the mycobacterial growth indicator tube (MGIT) culture system. Once the cultures flagged positive, Ziehl Neelsen staining was used to confirm the presence of acid-fast bacilli (AFB). All samples where AFB were present in the absence of cording were identified with GenoType^®^ Mycobacterium CM regardless of specimen type. A total of 194 isolates were collected. After data cleaning, 11 isolates were excluded to prevent duplication, and the remaining 183 isolates were included in the study (Figure 1). Data cleaning and deduplication were performed according to the following criterion: if numerous isolates from the same patient had the same NTM identification, only the first isolate was included.

### Data collection and laboratory testing

Data collection and supplementary laboratory testing were conducted between September 2020 and September 2021. The NHLS Laboratory Information System was used to collect laboratory and demographic data for the 183 isolates comprising the study. This included GenoType^®^ Mycobacterium CM VER 2.0 results, specimen type, direct auramine smear microscopy findings, previous NTM results, prior *M. tuberculosis* diagnosis and susceptibility results, and demographic information related to age and gender.

GenoType^®^ NTM-DR Ver 1.0 was only performed on *M. avium, M. intracellulare, M. abscessus* complex, and *M. chelonae* (*n* = 145). It was not performed on 38 isolates, which belonged to species beyond the scope of the assay’s intended purpose. GenoType^®^ NTM-DR was strictly performed according to the manufacturer instructions.

### Data analysis

Data analysis was performed by the Department of Biostatistics, Faculty of Health Sciences of the University of the Free State, using SAS Software, version 9.4 (SAS Institute Inc., Cary, NC, USA). Continuous variables were summarised by medians, minimum, maximum, and interquartile range (IQR). Categorical variables were summarised by frequencies and percentages. Differences between groups were evaluated using appropriate statistical tests (Chi-Square or Fisher’s Exact Test). Statistical significance was set at *p* < 0.05.

### Ethical considerations

Ethics approval was obtained from the Health Sciences Research Ethics Committee (HSREC) at the University of the Free State (UFS-HSD2020/1244/2909), the Environment and Biosafety Research Ethics Committee (EBREC) at the University of the Free State (UFS-ESD2020/0098), and the Universitas Academic Hospital NHLS Business Manager.

## Results

### Demographics and specimen type

In total, 183 isolates were identified from 182 patients. The median age of the patients was 36 years (range 1 day to 81 years; IQR: 24–50 years). Of the 183 isolates, 114 (62.3%) were from male patients, while 68 (37.2%) were from female patients. The gender of the patient of one isolate was unknown. Sputum was the most common specimen, *n* = 155 (84.7%), followed by gastric aspirate, sterile fluid, and cerebrospinal fluid (Table 1).

**TABLE 1:** Frequency of specimen types from where non-tuberculous mycobacteria were cultured (*n* = 183).

Specimen type	*n*	%
Sputum	155	84.7
Gastric aspirate	12	6.6
Sterile fluid	10	5.5
Cerebrospinal fluid	3	1.6
Abscess	2	1.1
Superficial swab	1	0.6

**Total**	**183**	**100.0**

*n*, number of isolates.

### Laboratory diagnosis and results

Only one specimen had AFB observed on auramine stain, from a patient who had repeatedly cultured *M. intracellulare* on 11 different occasions. The majority (87.4%) of the isolates (*n* = 160/183) were from patients who did not have NTM detected before. Twenty-three isolates (12.6%) were from patients who had previously cultured the same species. Of those 23, 15 (65.2%) isolates were from patients where the same NTM was isolated twice, while the remaining eight were from patients with the same NTM isolated three times or more. *M. intracellulare* was the most common species isolated (*n* = 19, 82.6%), followed by *M. abscessus* (*n* = 1), *M. scrofulaceum* (*n* = 1), *M. kansasii* (*n* = 1), and other *M. species* (*n* = 1). Of 183 patient isolates, 52 (28.4%) were from patients who previously had laboratory-confirmed TB.

### GenoType^®^ Mycobacterium CM identification

*Mycobacterium intracellulare* was the most common species (*n* = 140, 67.6%), followed by *M. fortuitum, M. species* (not speciated because of assay limitations), *M. kansasii*, and *M. gordonae,* while two different species were identified from 24 isolates. Thus, 207 species were identified from 183 isolates (Table 2). From these, GenoType^®^ NTM-DR was performed on 145 different isolates (Table 3).

**TABLE 2:** GenoType^®^ Mycobacterium CM identification of non-tuberculous species.

GenoType^®^ Mycobacterium CM result	*n*	%
*M. intracellulare* †	140	67.6
*M. fortuitum*	26	12.6
*M. species*	9	4.3
*M. kansasii*	8	3.9
*M. gordonae*	8	3.9
*M. scrofulaceum*	6	2.9
*M. tuberculosis*	4	1.9
*M. avium* †	3	1.4
*M. abscessus* †	1	0.5
*M. chelonae* †	1	0.5
*M. szulgai*	1	0.5

**Total**	**207**	**100.0**

*M, Mycobacterium; n*, number of isolates.

† , GenoType^®^ NTM-DR was only performed on these isolates as per the package insert.

**TABLE 3:** GenoType^®^ NTM-DR identification of non-tuberculous mycobacteria.

GenoType^®^ NTM-DR identification result	*n*	%
*M. intracellulare*	133	91.7
*M. species*	4	2.8
Uninterpretable	3	2.1
*M. avium*	3	2.1
*M. abscessus subsp. abscessus*	2	1.4
*M. chelonae*	0	0
*M. chimaera*	0	0

**Total**	**145**	**100.0**

*M, Mycobacterium; n*, number of isolates; NTM, non-tuberculous mycobacteria.

### GenoType^®^ NTM-DR identification

Of the 145 isolates identified with the GenoType^®^ NTM-DR assay (Table 3), the results were interpretable for 142 isolates, and the majority were *M. intracellulare* (*n* = 133, 91.7%) (Table 3). Initially, five results were uninterpretable. Two of them were resolved as *M. intracellulare* by re-culturing the isolate and repeating GenoType^®^ NTM-DR.

### Discordant identification results

**FIGURE 2:**
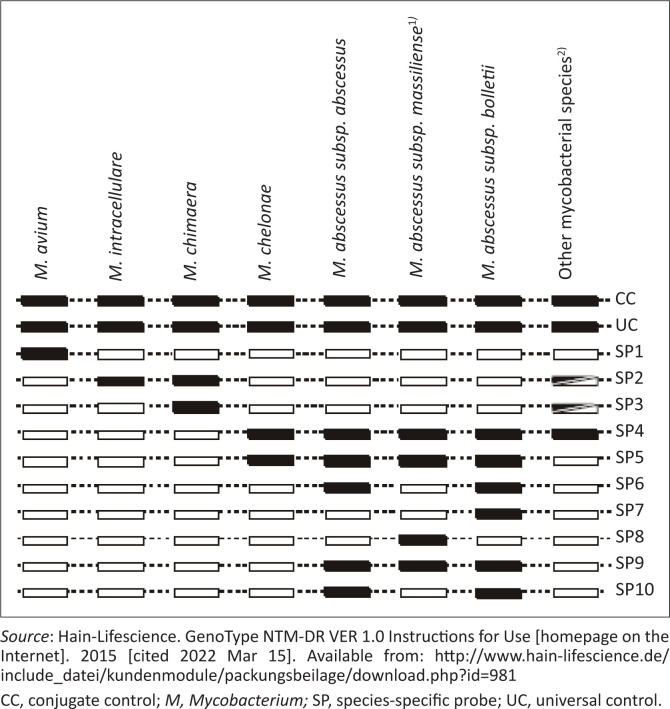
GenoType^®^ NTM-DR interpretation chart.

There were eight GenoType^®^ NTM-DR discordant identification results, three of which were uninterpretable (Table 4 and Figure 2). Isolate #88, #156, and #198 were mixed NTM cultures, as species-specific probes (SP) 2 and 4 were present, corresponding to *M. intracellulare* mixed with other mycobacterial species, respectively (Table 4). For both GenoType^®^ Mycobacterium CM and GenoType^®^ NTM-DR, the manufacturer’s instructions explicitly state that the presence of more than one species can hamper the interpretation of results.^15,23^

**TABLE 4:** Discordant non-tuberculous mycobacteria identification results.

Isolate number	GenoType^®^ Mycobacterium CM	GenoType^®^ NTM-DR	GenoType^®^ NTM-DR band pattern
#88	*M. intracellulare*	*M. species*	CC, UC, SP2 (dark), SP4 (faint)
#156	*M. intracellulare*	*M. species*	CC, UC, SP2 (dark), SP4 (faint)
#190	*M. intracellulare* *M. fortuitum*	*M. abscessus subsp. abscessus*	CC, UC, SP4, 5, 6, 9, 10
#198	*M. intracellulare* *M. gordonae*	*M. species*	CC, UC, SP2 (faint), SP4 (dark)
#199	*M. intracellulare* *M. gordonae*	*M. species*	CC, UC, SP4
#39	*M. intracellulare*	Uninterpretable	CC, UC
#56	*M. chelonae*	Uninterpretable	CC, UC, SP5
#62	*M. intracellulare*	Uninterpretable	CC

CC, conjugate control; *M, non-tuberculous;* SP, species-specific probe; UC, universal control; NTM, non-tuberculous mycobacteria.

For isolate #199, only SP4 was present, corresponding to other mycobacterial species, although *M. intracellulare* together with *M. gordonae* were identified with GenoType^®^ Mycobacterium CM. For isolate #190, GenoType^®^ NTM-DR was accepted as the correct result as the pattern of species-specific probes (SP4, 5, 6, 9, 10) is only found in *M. abscessus subsp. abscessus*. Regrettably, additional troubleshooting through the repetition of GenoType^®^ Mycobacterium CM tests or sequencing was not carried out because of cost and time constraints.

### Macrolide and aminoglycoside susceptibility

Regarding genotypic macrolide susceptibility, 137 (94.5%) of the 145 isolates were susceptible, four (2.8%) were resistant, and four (2.8%) were uninterpretable. Macrolide-resistant isolates included two *M. abscessus subsp. abscessus,* and two *M. intracellulare* strains.

GenoType^®^ NTM-DR was performed on 121 first-time isolates; two (1.7%) were macrolide resistant. GenoType^®^ NTM-DR was also performed on 20 of 23 recurrent isolates; two (10.0%) were macrolide resistant. There was an association between repeated isolation of the same NTM species and macrolide resistance; however, this was not statistically significant (*p* = 0.0965). An association was found between macrolide resistance and the female gender, which was statistically significant (*p* = 0.0258). Of the isolates from 57 female patients, four (7.0%) were macrolide resistant, whereas no macrolide resistance was detected in any isolate from male patients. No genotypic aminoglycoside resistance was detected, and six (4.1%) isolates were uninterpretable.

## Discussion

Worldwide, the prevalence of NTM varies according to patient demographics such as age, gender, and geographical location.^4^ The most common specimen in this study was sputum, followed by gastric aspirate and sterile fluid. This is important to note, as NTM cultured from non-sterile sites often reflects contamination or colonisation.^2^ The majority (62.3%) of isolates from our study were from male patients, in keeping with a study from Botswana, where 53% of isolates were from male patients.^26^ Furthermore, this correlates with TB in Africa and South Africa specifically, which also has a higher incidence in men than in women.^33^ Nevertheless, it is important to note that this correlation does not establish a direct causal linkage between the two phenomena. Instead, it might be explained by the confounding factor whereby men exhibit a higher TB prevalence than women, resulting in a greater submission of follow-up samples for mycobacterial culture, consequently yielding a higher frequency of NTM isolates among men relative to women.

The most common species in our study, in descending order, were *M. intracellulare, M. fortuitum, M. species, M. kansasii, M. gordonae*, and *M. scrofulaceum*. These species were predominately found in sputum, gastric aspirates, and fluid aspirates. These findings correlate well with previous studies from KwaZulu-Natal and Cape Town in South Africa, and Botswana, where the most common specimen and species were respiratory samples and *M. intracellulare,* respectively.^26,27,28^ It is important to highlight that *M. intracellulare* constitutes one of the species encompassed by MAC.^1^ *Mycobacterium kansasii* was also among the common species, as was seen in studies from KwaZulu-Natal and miners in the North West.^27,29,30^ Although *M. fortuitum* was the second most common species in the study, most of these were probably not clinically significant as it is of low virulence and is an uncommon cause of pulmonary disease. Furthermore, none of the *M. fortuitum* isolates was cultured more than once in this study, and many were from cultures mixed with *M. intracellulare*.^3,34^

Our study did not detect *M. chimaera.* This is important as numerous studies have found that *M. chimaera,* which is also a constituent of MAC, is frequently misidentified as *M. intracellulare*.^1,16,35,36^ While no prior investigations in South Africa have investigated *M. chimaera*, likely because of the limitations of GenoType^®^ Mycobacterium CM/AS used in local laboratories, it is improbable that *M. chimaera* was overlooked in our study as prior research has demonstrated the reliable identification of *M. chimaera* using GenoType^®^ NTM‑DR.^35^

Direct auramine staining was positive in only one isolate included in the analysis. This isolate was from a patient that cultured the same NTM 11 occasions, strongly suggesting clinical disease. The observed low positivity rate is anticipated, given the test’s characteristics, as culture is much more sensitive. Various factors influence sensitivity, including specimen type, disease prevalence, collection precision, organism concentration, and laboratory processes.^12^ Although auramine microscopy has poor sensitivity and specificity, it has been shown that a positive auramine, combined with a negative Xpert MTB/RIF, has an excellent specificity and positive predictive value (PPV) of 97.0% and 95.8%, respectively.^14^ In our study, the patient who cultured *M. intracellulare* on 11 different occasions had a positive auramine and numerous negative Xpert Ultra results, which strongly indicate disease.

Given the lack of clinical and radiological information, we could not definitively confirm the clinical significance of these NTM isolates. However, a study from Croatia found that using the American Thoracic Society/Infectious Diseases Society of America (ATS/IDSA) microbiological criteria alone, in their setting, had a PPV value of 59.8%, while using stricter microbiological criteria (same NTM ≥ 3) had a PPV of 93.3%.^37^ Another factor that increases the likelihood of clinical disease is isolates from sterile sites.^3^ In our study, most isolates were cultured from sputum, which is not a sterile specimen. However, when using the ATS/IDSA microbiological criteria for NTM-PD and isolates from sterile sites as a surrogate for clinical significance, 37 of 183 isolates (20.2%) were likely clinically significant. This rate is consistent with studies from the UK and sub-Saharan Africa where 25% and 27.9% of NTM isolates, respectively, were clinically significant.^9,11^

A notable proportion, 28.4%, of the NTM isolates in the study were from patients with previous laboratory-confirmed TB. While prior PTB is a well-known risk factor for developing NTM-PD, it is pertinent to highlight that the isolates in this study emanated from patients being investigated for TB, and most specimens were not from sterile sites.^6^

Macrolide resistance remains low, detected in four of 145 isolates (2.8%), while aminoglycoside resistance was not detected. Our study found an association between female gender and macrolide resistance (*p* = 0.0258) as all four cases of resistance were detected in females. However, the sample size is too small to make definite conclusions, and males were predominant in this study. Although we could not find any literature that showed an association between female gender and NTM resistance, there are numerous studies that have reported NTM-PD in elderly thin females with no apparent risk factors, also known as the Lady Windermere syndrome.^38^ Nonetheless, the four resistant isolates in our study were from younger women.

Of the macrolide-resistant isolates, two were *M. intracellulare,* and two were *M. abscessus subsp. abscessus*. Both *M. abscessus subsp. abscessus* isolates had inducible macrolide resistance encoded by a functional *erm*(41) gene where the *erm*(41)T28 bands were present. This is expected as most *M. abscessus subsp. abscessus* have a functional *erm*(41) gene resulting in inducible macrolide resistance.^20,39^ Of the two macrolide-resistant *M. intracellulare* isolates, one was from a patient who had cultured *M. intracellulare* on 11 different occasions and had an absent *rrl* wild type band with a corresponding *rrl* MUT3 band indicating an A2059C mutation. This isolate likely acquired resistance because of macrolide exposure, as *rrl* mediated resistance is selected during macrolide-based therapy.^19,40^ This finding is also supported by Wetzstein et al.^18^ and Renvoisé et al.,^19^ who found that patients receiving treatment for NTM have higher rates of resistance to macrolides than treatment-naïve patients. The other *M. intracellulare* isolate had an absent *rrl* wild type band with no corresponding mutation band present. This could indicate an A2058T or A2059T mutation reflecting resistance or a silent mutation.^23^ However, the above findings can only be confirmed by performing phenotypic antimicrobial susceptibility testing or *rrl* gene sequencing and a more in-depth analysis of patient records and clinical data.

### Strengths and limitations

To our knowledge, this is the first study documented in South Africa that investigated antimicrobial susceptibility in NTM. This study is retrospective, laboratory-based and limited in size and setting. There were no clinical and radiological results of the patients to confirm NTM disease. Discordant results, including isolates that had band patterns that were of low intensity, were not confirmed with sequencing. The molecular test used for antimicrobial susceptibility testing did not test for all resistance mechanisms and was limited to macrolides and aminoglycosides. Lastly, the study was undertaken in early 2020 during the disruptive healthcare conditions of the coronavirus disease 2019 (COVID-19) pandemic, potentially affecting health-seeking behaviour and, consequently, our sample size.

## Conclusion

*Mycobacterium intracellulare* is the most common NTM species identified, followed by *M. fortuitum, M. kansasii, and M. gordonae.* Although susceptibility to macrolides and aminoglycosides remains high, nationwide surveillance of NTM disease and monitoring of resistance trends are important to guide future disease management.
